# The role of the primary cilium in thyroid function and dysfunction with implications for thyroid disease

**DOI:** 10.1007/s00418-025-02432-y

**Published:** 2025-11-06

**Authors:** Inés Martín-Lacave, Victoria Vázquez-Román, Beatriz Pérez-Fernández, José María Fernández-Santos

**Affiliations:** Departamento de Citología e Histología Normal y Patológica, Facultad de Medicina, Universidad de Sevilla, Av. Sánchez Pizjuán S/N, 41009 Seville, Spain

**Keywords:** Primary cilium, Thyrocytes, Thyroid cancer, Thyroid follicles, Thyroid hormone biosynthesis

## Abstract

The thyroid gland is a unique endocrine organ, composed of morpho-functional units called thyroid follicles, which are responsible for thyroid hormone (TH) biosynthesis, an iodination process demanding a highly oxidative yet protected environment. Despite primary cilium (PC) being observed in the thyroid gland more than a century ago, its precise role in thyroid activity remains rather unexplored. Given its strategic position at the apical surface of follicular epithelium, projecting into the lumen, PCs are crucial for the regulation of TH biosynthetic processes. Consequently, changes in thyroid function, either physiological or pathological, are reflected in PC characteristics. Similarly, defects in ciliogenesis are expected to lead to different pathological thyroid alterations. This review summarizes the current understanding of PC’s involvement in regulating normal thyroid activity and its modifications in functional and neoplastic thyroid diseases. Particular focus will be given to the notable loss of PCs in certain types of thyroid cancer and the promising potential of their restoration as a tumor suppressor strategy in thyroid tumorigenesis.

## Introduction

Thyroid hormones (THs) are unique among all hormones as they are the only ones that are iodinated. The iodination process of the precursor molecule, thyroglobulin (Tg), requires an extremely oxidative environment from which the thyroid must be protected. Therefore, the thyroid gland is a unique endocrine gland, as its major endocrine component is organized into completely closed compartments—the thyroid follicles—which are spheric-like arrangements of variable diameter. Thyroid follicles are lined by a well-polarized single epithelial layer, the follicular epithelium, composed of follicular cells or thyrocytes, which surrounds a central lumen where the glycoprotein Tg is secreted and stored in the colloid. Tg iodination and coupling processes take place extracellularly at the apical surface of thyrocytes, ensuring that these delicate biosynthetic processes are safely isolated in the follicular lumen.

Even though the thyroid gland was one of the organs where the primary cilium (PC) was first described more than a century ago (Zimmermann 1898), our understanding of its function within thyroid follicles remains limited. Given its strategic location at the interface between the apical surface of thyrocytes and the colloid, the PC must play a crucial role in TH biosynthesis. Therefore, alterations in thyroid functional status are expected to manifest in the characteristics of PCs, both in healthy thyroid tissue and in pathological or neoplastic conditions.

This review will first outline the general features of the PC, followed by a description of thyroid architecture and histophysiology. We will then examine the characteristics and potential functions of the PC in thyroid follicle activity. The review will conclude by summarizing the current understanding of PC’s involvement in various thyroid diseases, focusing particularly on the notable loss of PCs in some thyroid cancers and the promising potential of their restoration as a tumor suppressor strategy in thyroid tumorigenesis.

## Revisiting the structure and functions of the primary cilium

### Structure of the primary cilium

The PC is a cellular organelle whose relevance to the scientific community has changed over the years. It was first described in mammals by Zimmermann (1898), who referred to it as the “central flagellum” or “single cilium” (Zimmermann 1898). This researcher highlighted the association of the central flagellum with the distal centriole of the diplosome, differentiating them from motile cilia in multicilliated cells (Satir 1995) and, most importantly, correctly predicted their sensory function. However, it took more than a century for this hypothesis to be confirmed (Praetorius and Spring 2003).

After a period of scarcity of publications on central flagellum, the development of transmission electron microscopy (TEM) in the 1950s enabled the resurgence of research on cilia. However, many researchers were unaware of the prior literature on central flagella and, consequently, considered them rudimentary or vestigial organelles. Particularly, Sorokin (Sorokin 1968), who renamed the structure as “primary cilium,” diverging from Zimmermann’s initial more appropriate name. Nonetheless, the term “primary cilium” did not achieve widespread usage within the scientific community until the late twentieth century, following the identification of intraflagellar transport (IFT) in *Chlamydomonas* flagella (Kozminski et al. 1993). Subsequently, investigation of the PC has become a significant area of focus in modern scientific research (Bloodgood 2009).

Along with the technological advances in TEM, different types of cilia were described in eukaryotic cells, according to their ultrastructural and functional characteristics (Amack 2022). Precisely, on the basis of their motility and axonemal architecture, cilia are categorized into motile cilia, nodal cilia, kinocilia, and primary cilia (PCs) (Carvalho-Santos et al. 2011).

**Fig. 1:**
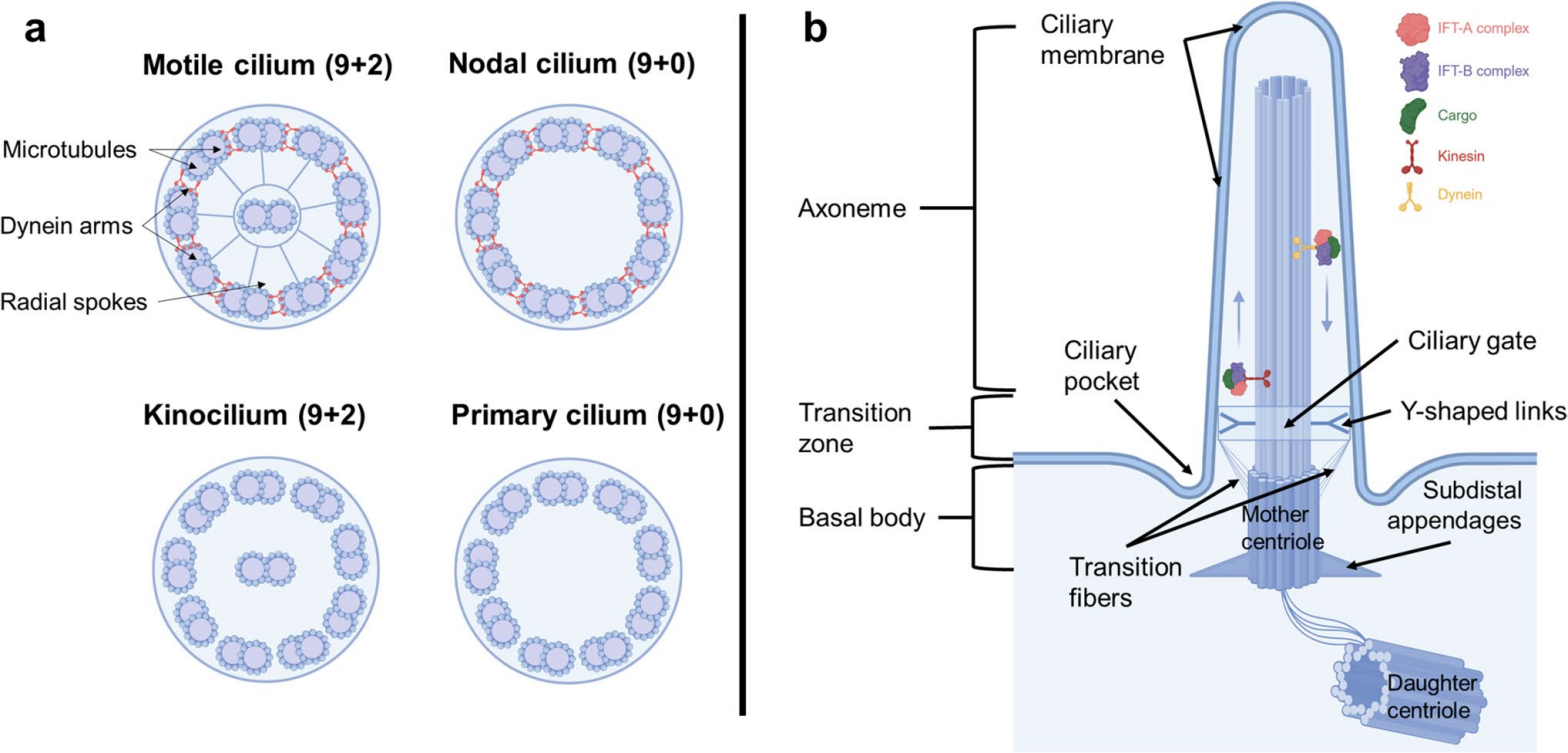
Structure of the different types of cilia described in eukaryotic cells. **a** Schematic cross sections of each type of cilia: motile cilium, with 9 + 2 microtubule arrangement and dynein arms; nodal cilium, with a 9 + 0 microtubules structure but is motile owing to the presence of dynein arms; kinocilium, with a 9 + 2 microtubules pattern and absence of dynein arms; and primary cilium, a non-motile cilium characterized by a 9 + 0 microtubule structure without dynein arms. **b** Schematic representation of different regions of the primary cilium, including the protein machinery involved in intraflagellar transport (IFT-A, IFT-B). Drawings made with Biorender and modified from Amack 2022 with permission from John Wiley and Sons

Motile cilia, found on the apical surface of multiciliated epithelial cells, have a 9 + 2 microtubule arrangement with dynein arms and radial spokes, enabling coordinated beating (Fig. 1a) (Nicastro et al. 2006). Nodal cilia, considered a motile subtype of PCs, are essential for embryonic development and left–right axis determination. They exhibit a 9 + 0 axoneme with dynein arms but lack radial spokes, resulting in a rotational beating pattern (Fig. 1a) (Hamada 2020; Amack 2022; Messmore et al. 2024). Kinocilia, although non-motile, possess a 9 + 2 structure and are involved in establishing planar cell polarity during inner ear hair cell development (Fig. 1a) (Zheng and Holt 2021). Finally, the PC is a non-motile organelle composed of four key regions that are described below (Fig. 1 a, b).

The basal body anchors the cilium to the cell via rootlets and connects to the plasma membrane through transition fibers (Elliott and Brugmann 2019). It originates from the older “mother” centriole, a barrel-shaped structure with nine microtubule triplets and distal/subdistal appendages. This structure serves as the microtubule-organizing center for axoneme formation (Breslow and Holland 2019; Hall and Hehnly 2021).

The transition zone is located between the basal body and axoneme, it acts as a selective barrier or “ciliary gate,” regulating protein trafficking into and out of the PC (Kobayashi and Dynlacht 2011; Reiter et al. 2012). It contains transition fibers and Y-shaped linkers that connect axonemal microtubules to the ciliary membrane, playing a critical role in ciliogenesis (Garcia-Gonzalo and Reiter 2017; Jensen and Leroux 2017; Osinka et al. 2019).

The axoneme is a cylindrical structure with a 9 + 0 microtubule arrangement, lacking dynein arms, which extends from the cell surface and forms a specialized ciliary membrane (Satir and Christensen 2007). The organization of the microtubules in the axoneme determines whether the cilium is motile or non-motile (Smith and Yang 2004). Extension of the ciliary axoneme requires intraflagellar transport (IFT), which describes the bidirectional transport of particles or cargos along microtubules (Sorokin 1968; Kozminski et al. 1993; Amack 2022). Anterograde transport is carried out by IFT-B complexes and kinesin-2 motors. Retrograde transport is carried out by IFT-A complexes and dynein motors. Disruptions in IFT components can impede ciliogenesis and result in dysmorphic cilia (Pazour et al. 2000; Pazour and Rosenbaum 2002; Tran et al. 2008; Cortellino et al. 2009). Extension of the axoneme is stabilized by post-translational modifications of tubulin subunits such as acetylation, de-tyrosination, polyglutamylation, and polyglycylation (Redeker et al. 1994; O’Hagan and Barr 2012).

The ciliary membrane is a specialized membrane that encloses the axoneme and, despite being continuous with the cell membrane, has distinct structural components (Jensen and Leroux 2017). The particular lipid composition of the PC membrane allows for preferential localization of receptors and osmotic capabilities (Nauli et al. 2003; Garcia-Gonzalo et al. 2015; Garcia-Gonzalo and Reiter 2017), for example, phosphatidylinositol 4-phosphate (PI4P), which supports the localization of certain G protein-coupled receptors or channels to the PC from many signaling pathways, conferring the PC the ability to function as a “cellular antenna” (Schneider et al. 2005; Elliott and Brugmann 2019). Some PCs also feature a ciliary pocket at their base—a membrane invagination linked to the actin cytoskeleton—important for membrane trafficking and protein import (Mill et al. 2023).

### Identification of the primary cilium in different cell types

As previously noted, parallel to advancements in TEM, during the 1960s to 1980s, a progressive increase was observed in the number of cell types in which the presence of a single cilium was detected. For example, in chronological order, cilia were described in adenohypophysial cells (Barnes 1961), smooth muscle cells (Sorokin 1962), Schwann cells (Grillo and Palay 1963), neurons (Allen 1965), fibroblasts (Wheatley 1969), and pancreatic endocrine cells (Boquist 1970), among others.

Nevertheless, PC description by TEM is very labor intensive and has inherent limitations. In fact, it has been estimated that unequivocal evidence of a PC is only likely to be present in four to five sections of the several hundred required to serially section a whole cell (Wheatley et al. 1996). Such improbability of seeing a PC underestimates their frequency within a given population when cells are analyzed in this way. Therefore, the identification of specific PC markers by immunofluorescence (IF) and confocal microscopy represented a significant advancement for studying the localization and frequency of PCs in different tissues (Wheatley et al. 1996; Poole et al. 1997). The most widely used marker for cilia among biologists has been acetylated α-tubulin, which stains the axoneme. However, a growing number of antibodies against various PC components are emerging. According to Hua and Ferland (Hua and Ferland 2017), these antibodies may be classified into three distinct groups, as follows:

(1) *Tubulin structural markers*, such as acetylated α-tubulin (Piperno and Fuller 1985), detyrosinated tubulin (Wheatley et al. 1994), and polyglutamylated tubulin (Wolff et al. 1992). (2) *Axoneme-associated proteins*, such as the proteins IFT20, IFT88 (Follit et al. 2009), and CSPP1 (Patzke et al. 2010). (3) *Membrane-bound cilia proteins*, such as ARL13B (Kasahara et al. 2014) and ACIII (Bishop et al. 2007), and transmembrane receptors such as somatostatin receptor 3 (SSTR3), which is characteristic of neuronal PCs but also localized in some endocrine tissues (Iwanaga et al. 2011). However, we also would include a fourth category of cilia markers, specifically, *basal body markers*, such as γ-tubulin (Komarova YuA et al. 1997) and pericentrin (Miyoshi et al. 2006).

Thanks to the development of these techniques, PCs have transitioned from being considered rudimentary organelles to being recognized as ubiquitous sensory cellular structures involved in many normal processes as well as in different pathological disorders (Wheatley 2004, 2018). Although these highly conserved structures are found across a broad range of species, a nearly ubiquitous appearance is observed only in vertebrates.

### Functions of the primary cilium

Numerous studies support the PC’s role in transducing several sensory signaling pathways, including hedgehog (Hh), Wnt, Hippo, calcium, and receptor signaling, as key receptors for these pathways localize to the PC’s membrane (Amack 2022). PCs sense a variety of extracellular signals and mediate crucial processes such as division, migration, cell proliferation, and tissue differentiation (Sorokin 1968; Tran et al. 2008; Higginbotham et al. 2012). Furthermore, these organelles are essential for maintaining tissue homeostasis, regulating cellular metabolism (Lee and Hughes 2023). Furthermore, PCs modulate autophagy through signaling pathways, including mammalian target of rapamycin (mTOR) and AMP-activated protein kinase (AMPK). Autophagic machinery is localized within the periciliary compartment, and a bidirectional interplay exists between PC dynamics and the autophagy–lysosome system (Pampliega et al. 2013; Orhon et al. 2015).

The role of the PC in sensory organs such as vertebrate retina (Chen et al. 2021; Barnes et al. 2021), olfactory sensory cells (Mukhopadhyay et al. 2008; Su et al. 2009), and mammalian vestibular cells (Caprara and Peng 2022) is well established. Strategically, the PC can be considered a “cellular antenna” capable of receiving and integrating extracellular and intracellular information (Picon-Galindo et al. 2022; Kalot et al. 2023; Mill et al. 2023). Nevertheless, PCs also perform a sensory function in diverse non-sensory cell types including neurons (Lee and Gleeson 2010), pancreatic islet (Lodh et al. 2014; Lodh 2019; Lee and Hughes 2023), chondrocytes (Yuan and Yang 2016; Tao et al. 2020), fibroblasts (Wheway et al. 2018), and epithelial cells of the oviduct (Abdelhamed et al. 2018; Hunter et al. 2024).

Moreover, the PC is a well-established mechanosensor whose rigidity, length, and membrane ion channels enable mechanotransduction in multiple cell types (Malone et al. 2007; Spasic and Jacobs 2017; Ma et al. 2017). Specifically, in renal tubular cells, this function relies on the polycystin-1/2 (PC1/2) complex, which increases intracellular Ca^2+^ and activates signaling cascades (Pazour and Rosenbaum 2002; Praetorius and Spring 2003, 2005; Satir and Christensen 2007; Spasic and Jacobs 2017, 2018). Similarly, bile flow in cholangiocytes triggers Ca^2+^ increase via polycystins and reduces cAMP through adenylate cyclase 6 (AC6) (Masyuk et al. 2006, 2008; Mansini et al. 2018), while in osteocytes, PC1/PC2, AC6, and transient receptor potential cation channel, subfamily V, member 4 (TRPV4) mediate mechanotransduction (Spasic and Jacobs 2017; Moore et al. 2018; Verbruggen et al. 2023).

### Cilia-related disorders: ciliopathies

As previously mentioned, the PC is essential for regulating signaling pathways during development and adult homeostasis. In humans, mutations in cilia-related genes cause a broad spectrum of more than 30 disorders, referred to as ciliopathies, which can affect many organs and systems (Badano et al. 2006; Fliegauf et al. 2007). Impaired primary ciliogenesis because of these mutations may result not only in the impoverishment of signaling cascades that course through PCs but also in the incorrect activation of signaling responses that are normally kept under control by a functioning ciliary axis (Mill et al. 2023).

Among disorders associated with PC dysfunction, the most studied and documented are those that affect the liver, pancreas, and kidney. In liver, they are properly called cholangiociliopathies as the only epithelial component of this organ showing PCs are cholangiocytes (Masyuk et al. 2008). Cholangiociliopathies result in cystic and/or fibrotic liver diseases due to decreased Ca^2+^ concentration and increased cAMP, leading to cholangiocyte hyperproliferation and abnormal liver function (Masyuk et al. 2006).

Faults in the PC may also affect normal metabolism, including diabetes mellitus, through the disruption of hormonal signal transduction and insulin release. Research demonstrating a correlation between mutations in genes such as *NEK8* and *IFT88* and defects in pancreatic β-cell PCs indicates their involvement in glycemic homeostasis regulation (Lee and Hughes 2023).

In the kidney, mutations in the genes encoding proteins PC1 and PC2 (*PKD1* and *PKD2*), which are localized to the PC, lead to a pathology called polycystic kidney disease (PKD). In most cases, these mutations have an autosomal dominant inheritance (ADPKD). ADPKD represents the most prevalent hereditary cystic kidney disease, marked by the progressive formation of fluid-filled renal cysts that compromise renal function, frequently culminating in end-stage renal disease and requiring dialysis or renal transplantation (Nauli et al. 2003; Ma et al. 2013, 2017).

## Thyroid architecture and histophysiology

### Epithelial components of the thyroid gland

The thyroid gland, a singular endocrine organ, is mainly composed of thyroid follicles. They consist of a single layer of follicular cells or thyrocytes organized as a polarized cuboidal epithelium, enclosing a central lumen, the follicular lumen. This lumen contains the secretory product of the follicular cells, the colloid, rich in the iodinated glycoprotein Tg, which is stored extracellularly in significant quantities. The follicular epithelium is surrounded by a basal lamina and reticular fibers. A network of nerve fibers and blood vessels, including fenestrated capillaries, can be observed in the connective tissue among thyroid follicles. Serving as the structural and functional units of the thyroid gland, thyroid follicles exhibit independent self-regulatory mechanisms, contributing to the inherent heterogeneity of the tissue (Colin et al. 2013).

Although follicular cells constitute the predominant endocrine cell population of the thyroid gland, they are not its sole epithelial component. C cells and ultimobranchial remnants, though present in significantly fewer numbers, are scattered among the thyroid follicles in the central area of the thyroid lobes. These three distinct cellular elements share a common endodermal origin, albeit from different embryonic regions. Evolutionarily, in mammals, C cells migrate from the ultimobranchial bodies to their definitive parafollicular location. This migration establishes a paracrine regulatory influence on follicular cells, in addition to their known function as calcitonin-producing cells involved in calcium homeostasis (Fernández-Santos et al. 2012; Johansson et al. 2015; Kameda 2016).

### Thyroid follicles: exclusive compartments for iodinated-TH biosynthesis

Follicular cells are responsible for producing and releasing the active THs thyroxine (T_4_) and triiodothyronine (T_3_). THs are iodinated compounds, and the essential trace component iodine, which comprises more than 50% of the molecular mass of T_3_ and T_4_, is a critical element of THs owing to its scarcity in the diet. Moreover, hormone iodination (iodine organification) is a complex process in which the generation of H_2_O_2_ and other dangerous oxidative species are involved.

As an evolutionary response to these two challenges, the thyroid tissue is organized into angiofollicular units (AFUs), as described by Colin et al. (Colin et al. 2013). The AFU is an independent unit composed of a single thyroid follicle together with its plexus of fenestrated capillaries. The AFU is an advanced system able to store iodine and THs to be used in long low-iodine dietary supply periods and, also, a structure where highly oxidative and putatively hazardous reactions are isolated and kept under control. Nonetheless, the thyroid gland is the endocrine organ with the highest rate of DNA mutations and cancer (Driessens et al. 2009).

**Fig. 2:**
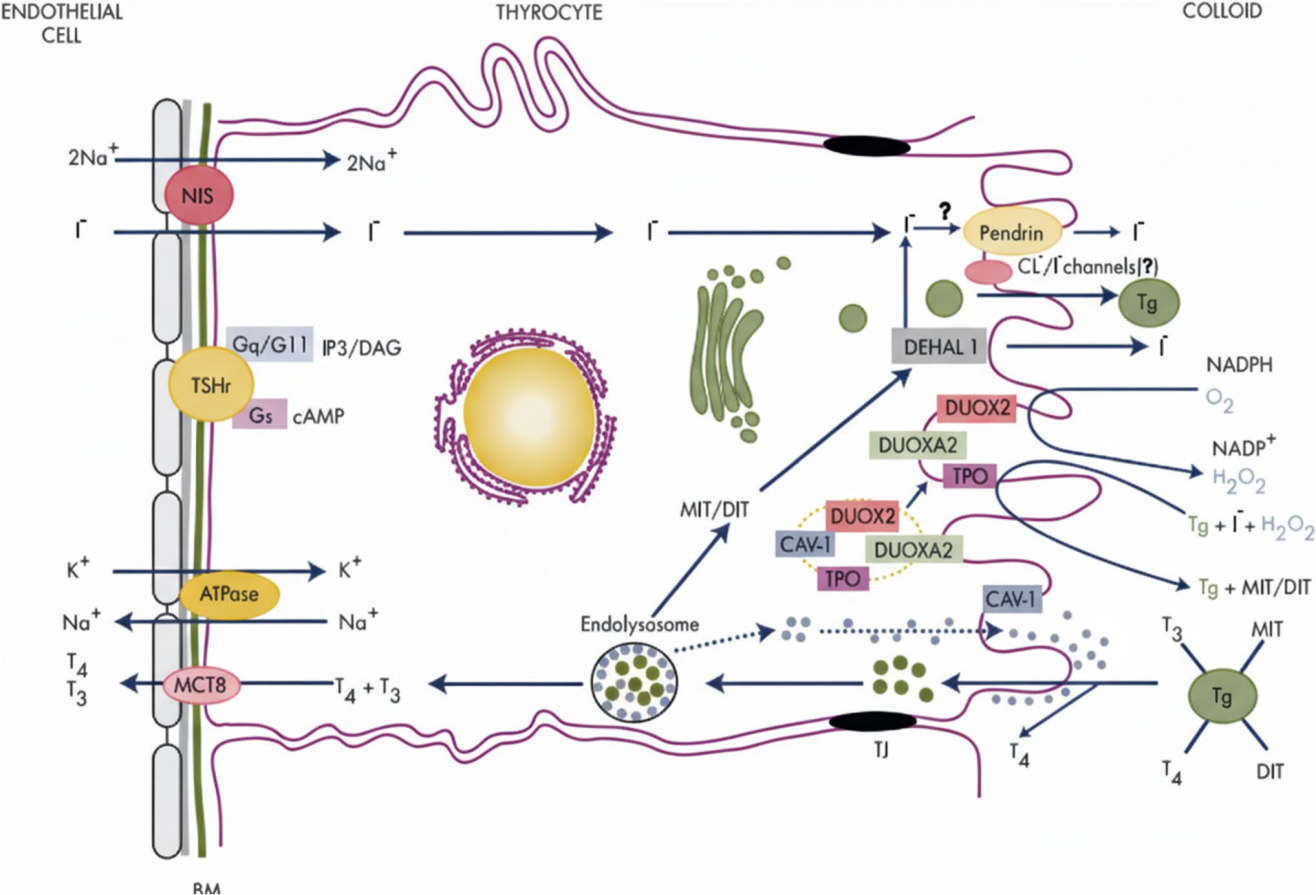
Schematic representation of the TH biosynthetic process by Colin et al. (2013), used with permission of Oxford University Press. *BM* basal membrane, *DAG* diacylglycerol, *DEHAL* Iodotyrosine dehalogenase 1, *DIT* diiodotyrosine, *DUOX2* dual oxidase 2, *DUOXA2* DUOX maturation factor 2, *Gq/G11* guanine nucleotide-binding protein αq and α11 subunits, *Gs* guanine nucleotide-binding protein α-subunit, *IP3* inositol triphosphate *MIT* monoiodotyrosine, *Tg* thyroglobulin, *TJ* tight junctions, *TPO* Thyroperoxidase, *TSHr* TSH receptor

The very complex process of TH synthesis in the thyrocyte consists of the stages described below; key players and the main biosynthetic steps are depicted in Fig. 2:

1. *Iodine uptake*. Follicular cells are one of the few cells able to extract iodide (I^−^) from plasma. They obtain and concentrate it in the thyroid gland where I^−^ is found at a concentration 20–50 times higher than in blood. Its entry into the cell through the basolateral membrane is based on an active transport mechanism. This process is mediated by the Na^+^/I^−^ symporter (NIS), which binds Na^+^ in the presence of I^−^, forming a complex that translocates one I^−^ and two Na^+^ into the cell (Dai et al. 1996; Portulano et al. 2014). The regulation of NIS, both at the transcriptional and post-transcriptional levels, is carried out by thyroid stimulating hormone (TSH), which, through a thyroid-specific mechanism involving cAMP as a secondary messenger, stimulates the expression of the NIS gene (Kogai et al. 1997). Further, at the post-transcriptional level, TSH regulates iodine uptake by mediating the subcellular distribution of NIS. In the absence of TSH, NIS redistributes from the basolateral membrane to the intracellular compartment, reducing I^−^ uptake (Riedel et al. 2001; Dohán et al. 2003; Riesco-Eizaguirre and Santisteban 2006). There is a negative regulation mechanism for NIS expression. In cases of high plasma I^−^concentration, there is a decrease in I^−^ uptake and organification, known as the Wolff–Chaikoff effect, which is mediated by a decrease in NIS expression (Eng et al. 1999). The passage of I^−^ into the lumen through the apical membrane of the thyrocyte is carried out by poorly understood transporters such as CLC-5 (Senou et al. 2010), anoctamin-1 (Twyffels et al. 2014), CFTR (Devuyst et al. 1997), and pendrin, named after the Pendred syndrome gene (PDS) (Royaux et al. 2000).

2. *Tg synthesis.*. Tg is a glycoprotein synthesized and released exclusively by follicular cells and stored in the colloid of the thyroid follicle. Initially, Tg molecules remain in a soluble form at the periphery of the lumen. Subsequently, they gradually migrate to the center of the follicle, where they are stored as highly concentrated, insoluble, covalently cross-linked, multimerized Tg globules. This process enables the follicle to store large quantities of Tg (Gerard et al. 2004). The regulation of Tg expression is primarily carried out by TSH via the cAMP/protein kinase A (PKA) pathway, although insulin-like growth factor 1 (IGF-1) and insulin have been shown to significantly increase Tg mRNA levels (Santisteban et al. 1987).

3. *Iodine organification*. Once I^−^ is concentrated in the follicle lumen, it rapidly undergoes an oxidation process, and it is incorporated into the Tg molecule, forming monoiodotyrosine (MIT) and diiodotyrosine (DIT). This oxidation reaction is catalyzed by thyroperoxidase (TPO) (Senou et al. 2009). This reaction is dependent on H_2_O_2_ (Song et al. 2007; Poncin et al. 2009; Ohye and Sugawara 2010), which is produced by a mechanism involving an enzymatic system of NADPH oxidases called DUOX1 and DUOX2 (for dual-oxidase), located in the apical membrane of the cell alongside TPO (Rigutto et al. 2009; Carvalho and Dupuy 2017; Poncelet et al. 2019). TPO activity is inhibited by propylthiouracil (PTU) and methimazole (MMI), which have been used as antithyroid drugs for decades (Cooper 2005). MIT and DIT finally combine to form T_3_ and T_4_ within the Tg molecule by an ether bond between two tyrosine molecules through a process called coupling (Virion et al. 1985). The control of the expression and activity of this entire enzymatic machinery is regulated by TSH, which, through cAMP, stimulates the production of H_2_O_2_, as well as the oxidation and incorporation of I^−^ into the Tg molecule (Gerard et al. 1988; Damante and Di Lauro 1994; Kambe and Seo 1997; Kogai et al. 1997). Iodine organification follows the principle formulated as “the last-come-first-served principle,” by which the iodination process preferentially involves newly-synthesized, soluble Tg molecules at the apical surface of follicular cells (Schneider 1964; Gerard et al. 2004).

4. *Tg internalization, processing, and TH release*. As previously described, the follicle lumen contains a dynamic pool of Tg, whose composition is highly dependent on factors such as hormonal and nutritional status, and iodine availability. This pool comprises both older, highly condensed, insoluble Tg and newly-synthesized, soluble Tg, present in varying proportions. As for iodine organification, these two types of Tg also follow the “last-come-first-served principle” for the internalization process. Subsequent steps of TH production include the internalization of the soluble Tg by the thyrocyte, followed by lysosomal digestion carried out by the action of hydrolytic enzymes, mainly cathepsins B, L, and D (Dunn et al. 1991b, a; Brix et al. 2020). Tg internalization involves fluid pinocytosis and receptor-mediated mechanisms that involve LRP2/megalin and ASGPR (Marinò et al. 2000b, a; Marinò and McCluskey 2000). In the case of TSH overstimulation or iodine deprivation, prior to endocytosis, a previous step of luminal digestion of condensed Tg occurs by cathepsins. Solubilized Tg then follows the same lysosomal path described above. After Tg digestion, T_3_ and T_4_ are released into circulation.

### Heterogeneity of thyroid follicles

Because of the “last-come-first-served principle,” explained above, the follicle lumen of every single follicle contains older non-soluble and newly-synthetized soluble Tg in very different proportions with large variations in iodine and THs. Tg acts as a potent inhibitor of the expression of specific genes participating in TH synthesis (Suzuki et al. 1999a, b, 2011). This negative feedback effect appears to depend on its degree of iodination (Huang et al. 2011) and has been proposed to be involved in thyroid follicle heterogeneity (Sellitti and Suzuki 2014). In fact, in normal thyroid tissue, small active follicles displaying high columnar polarized epithelium coexist with larger hypofunctioning follicles surrounded by low cuboidal or flattened thyrocytes; this heterogeneity in size and activity are hallmarks of the human thyroid gland (Faggiano et al. 2004).

The intrinsic variation in the morphology of thyroid follicles clearly increases with age. Specifically, Lee et al. (2016a) documented a spectrum of histological changes in thyroid follicles of aged women, including: a heterogeneous follicular size and enlargement, an increased morphological irregularity, an increase in the size or number of Tg globules, the presence of Sanderson’s polsters within the walls of enlarged follicles, the oncocytic transformation of follicular cells, and the observation of markedly dilated follicles devoid of luminal colloid. Comparable histological findings were reported in aged mice, despite the absence of significant changes in serum T_3_ levels (Lee et al. 2016a). It is plausible that this heterogeneity, coupled with other factors, such as the exceptionally prolonged follicular cell turnover time (Coclet et al. 1989), contributes to goiter development, particularly in environments characterized by fluctuating iodine availability.

## History of the primary cilium in the thyroid

### Discovery of the thyroid primary cilium

**Fig. 3:**
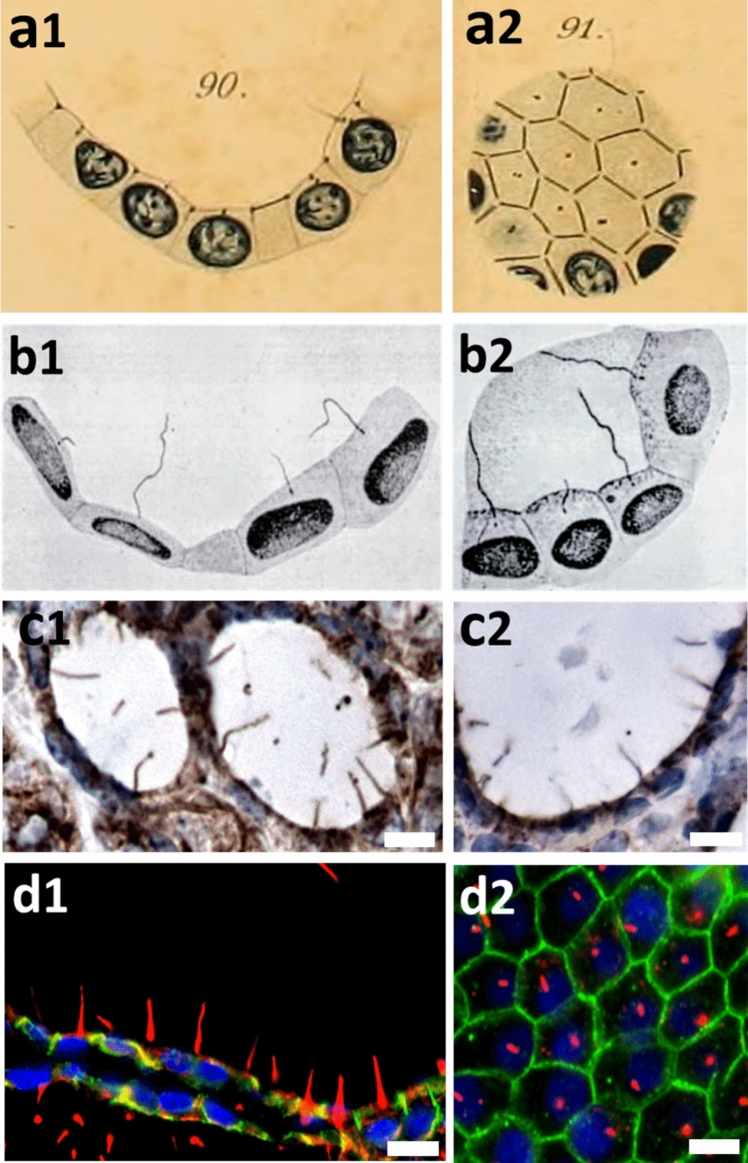
Identification of PC in vertebrate thyroid glands. **a1**, **a2** Original drawings of the PC (“central flagellum”) from human thyroid published by K.W. Zimmermann (1898), obtained from sections stained by Heidenhain’s hematoxylin method. **b1**, **b2** Original drawings of PCs from dogfish thyroid published by E.V. Cowdry (1921), obtained from sections stained by Cajal’s silver method. **c1**, **c2** Photomicrographs of normal human thyroid follicles immunostained by IHC using anti-acetylated α-tubulin, followed by the streptavidin–biotin-peroxidase method, and counterstaining with Harris hematoxylin. **d1**, **d2** Photomicrographs of normal human thyroid sections immunostained by IF using anti-acetylated α-tubulin for the axoneme (red) and anti-E-cadherin for the cell boundary (green). Nuclei were labeled with DAPI. As observed, there is almost complete correspondence between the original drawings sketched more than a century ago and the current images of PCs projecting from the follicular epithelium into the lumen (**a**, **b**). Scale bars: **d1** 2 µm; **c1**, **c2**, and **d2** 5 µm. (Images in **a** and **b **used with permission of Springer Nature and John Wiley and Sons)

As stated above, the human thyroid gland was one of the epithelial tissues in which Zimmermann described the “central flagellum” for the first time in mammals (Zimmermann 1898). The critical technical advance that made possible the discovery of the PC was the development of the iron-hematoxylin stain by Heidenhain in 1894 (Heidenhain 1894). Nevertheless, “anyone familiar with research on the central flagellum will concur with Zimmermann’s assertion, that ‘this structure is among the most optically difficult’ to study” (Bloodgood 2009). Zimmermann had also the merit of identifying the central flagellum as a special class of cilia distinct from the motile cilia observed in multiciliated cells and, most importantly, correctly predicted their sensory function. While this author published a remarkable collection of drawings illustrating diverse cell types exhibiting PCs (kidney tubules, pancreatic ducts, seminal vesicles, and thyroid), to the best of our knowledge, this review constitutes the first instance of Zimmermann’s thyroid drawings being reproduced (Fig. 3a).

The next mention of the presence of PCs in the thyroid gland was not until Cowdry described it in (1921) in dogfish, using a modification of the Cajal method. He revealed that each follicular cell was provided with a large flagellum which extended into the colloid substance (Cowdry 1921). Nevertheless, this author was apparently unaware of earlier publications that proposed a sensory role for central flagellum. Therefore, he erroneously concluded that this organelle was without apparent adaptive value, a sentiment echoed by subsequent researchers for decades. Despite this, his drawings of the dogfish thyrocytes displaying large flagella blackened by the silver method are striking (Fig. 3b).

### Description of the thyroid primary cilium by electron microscopy

Approximately 60 years elapsed after their discovery before PCs could be observed in detail by TEM in the thyroid gland of different vertebrates, such as birds (Fujita 1963; Muramoto 1964), fishes (Fujita and Honma 1966), amphibians (Setoguti 1973), and mammals (Tashiro and Sugiyama 1964). Specifically, in humans, Lupulescu et al. (1967) and Klinck et al. (1970) were the first authors to describe the occasional presence of a single cilium or two cilia per follicular cell protruding into the colloid, while meticulously characterizing the ultrastructure of the normal thyroid gland. They inferred that most follicular cells likely bore at least one cilium. Nevertheless, they also inaccurately described instances where cilia, when cross-sectioned, exhibited a motile-cilia-like pattern (9 + 2).

**Fig. 4:**
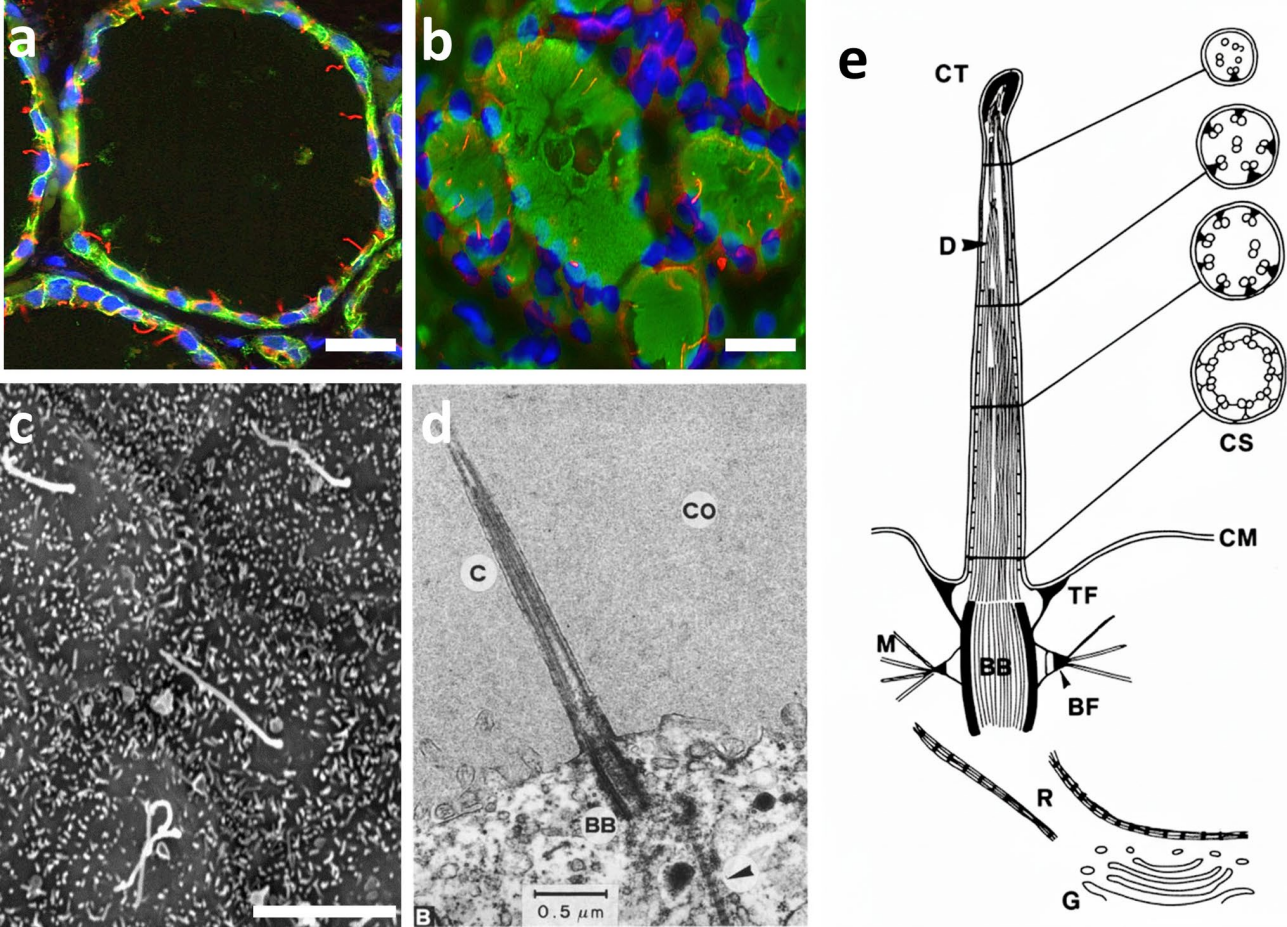
Morphology of the PC in human thyroid tissue. **a** Double IF with anti-acetylated α-tubulin (red) and E-cadherin (green) antibodies and counterstained with DAPI (blue). **b** Double IF with anti-acetylated α-tubulin (red) and Tg (green) antibodies and counterstained with DAPI (blue). **c** SEM micrograph. **d** TEM micrograph. **e** Schematic representation of the ultrastructure of the PC at TEM level. *BB* basal body, *BF* basal foot, *CM* cell membrane, *CS* ciliary cross-sections, *CT* ciliary tip, *D* doublets (unparallel arrangement), *G* Golgi apparatus, *M* microtubules, *R* rootlets, *TF* transitional fibers. As can be seen in all the pictures, at least one long PC extends up from the apical surface of every follicular cell into the colloid. Scale bars: **a** and **b** 25 µm, **c** 5 µm. (**d** and **e** adapted from Martin et al. 1988, with the permission of Springer)

The intrinsic limitations of TEM to observe the magnitude of ciliated follicular cells in human thyroids were eventually overcome by the studies of Sobrinho-Simões and Johannessen (Sobrinho-Simões and Johannessen 1981) using scanning electron microscopy (SEM). They demonstrated that most follicular cells had one to five cilia perpendicular to the central area of the cell membrane. Cilia had a rather uniform diameter, about 0.2 µm wide, but varied greatly in length, up to 6 µm. These authors referred to them simply as “cilia” and erroneously suggested that in thyroid tissue they might play a functional role by stirring the colloid (Sobrinho-Simões and Johannessen 1981) (Fig. 4c).

A detailed and definitive understanding of the ultrastructure and behavior of PCs in human thyrocytes, as well as the establishment of the nomenclature of “primary cilia” as introduced by Sorokin in 1968 (Sorokin 1968), would require the studies of Martin et al. (Martin et al. 1988) at the TEM level. These authors clarified previous contradictory reports and claimed that PCs of living cells were immotile and exhibited in cross-section a 9 + 0 microtubule arrangement at the base of the shaft. They also demonstrated that dynein arms, radial spokes, and central microtubules were absent (Fig. 4d, e). Consequently, they concluded that cilia of follicular cells were structurally typical PCs, like those found in the cells of practically all organs during ontogenesis or in adult organisms.

### Visualization of the thyroid primary cilium by immunofluorescence

Since the late 1990s, the presence of PCs has been increasingly described in various tissues through IF, leading to the current understanding that virtually all eukaryotic cells, at least at some point in their lifespan, possess a PC (Wheatley 2018). Specifically, regarding the thyroid gland, the first description of the PC by IF in follicular cells was provided by Utrilla et al. (2015) (Figs. 3d and 4a, b). A year later, Lee et al. also identified PCs in the human thyroid using double IF (Lee et al. 2016b). We have also attempted to visualize PCs by immunohistochemistry (IHC); however, due to their small size, they were only detectable in certain over-stained areas of thyroid sections under light microscopy (Fig. 3c).

## The primary cilium in the thyroid gland

### The primary cilium in mammalian thyroid

Ciliogenesis has been a subject of research in thyroid organogenesis and differentiation across a wide spectrum of vertebrate species, including fish, amphibians, reptiles, birds, and mammals (Rupik 2013). Specifically, TEM studies in different mammalian embryos have revealed that all cells involved in the formation of the follicular lumen possess a single cilium that extends laterally into the intercellular space (Calvert and Pusterla 1973; Rémy et al. 1980; Chan 1983; Johansson et al. 2021).

The most comprehensive insights into primary ciliogenesis during thyroid differentiation were provided by Lee et al., using a model of mice devoid of PCs generated by thyroid follicular epithelial-cell-specific deletion of the gene *Ift88* (Lee et al. 2019). They observed that PC loss from postnatal thyroid tissue of deficient mice, with increasing age, caused progressively dilated and destroyed follicles and, eventually, evolved to malignant transformation. Consequently, these authors suggested that PCs must play a crucial role in maintaining the structural integrity of thyroid follicles by acting on cell polarity (Lee et al. 2019).

In relation to the postnatal thyroid gland, Utrilla et al. investigated whether the existence of PCs in thyrocytes was a general event in different mammals, using both IF and electron microscopy (TEM and SEM). According to their results, PCs were present in the adult thyroid gland of most mammal species studied (human, pig, guinea pig, and rabbit), usually as a single copy per follicular cell. Strikingly, those organelles were apparently absent from murine thyroid tissue (Utrilla et al. 2015). Upon further examination of thyroid tissue from Wistar rats across various ages, the same authors observed that PCs were very short and scarce, having instead many centrioles lacking cilia located beneath the apical cell surface (Pérez-Fernández et al. 2024). Nevertheless, Lee et al. reported the presence of PCs in mice thyroids by double IF, employing antibodies against acetylated α-tubulin or ARL13B and γ-tubulin, though bona fide PCs exhibiting an axoneme were rarely displayed (Lee et al. 2019, 2021b).

### Frequency and length of primary cilia in normal human thyroid

As we have described so far, PCs are displayed by thyroid follicular cells from organogenesis through differentiation and during postnatal life. In contrast to multiciliated cells, cells with PCs are capable of undergoing mitosis, PCs being a postmitotic organelle that typically assemble when cells exit the cell cycle (Amack 2022). Therefore, the presence of PCs in follicular cells is contingent on their post-mitotic state. The thyroid gland is characterized by a relatively low cell turnover rate compared with other tissues, with an estimated average lifespan of human thyrocytes around 8.5 years/3100 days. A shorter turnover time was described in the young thyroid and in follicular cell nodules (Coclet et al. 1989). This would account for the relatively high percentage of ciliated follicular cells observed in normal human thyroids. Furthermore, it would also justify variations in the frequency of ciliated thyroid cells during different functional or pathological alterations of the gland, as will be discussed below.

The first data on the average frequency and characteristics of PCs in the normal human thyroid gland were reported by Sobrinho-Simões and Johannessen (1981) using SEM. As previously mentioned, this method is ideal for obtaining a panoramic view of the lumen of thyroid follicles and for detailed observation of the apical surface of the epithelium, although it has intrinsic methodological limitations. These authors reported that nearly all follicular cells were ciliated, with only 8.4% lacking cilia. Moreover, most follicular cells had one to five cilia, which were usually parallel to each other and perpendicular to the cell surface (Sobrinho-Simões and Johannessen 1981).

While formalin-fixed, paraffin-embedded sections may not offer the same level of detail as SEM, and potentially compromise PC integrity, this method allows for the examination of numerous samples with diverse pathologies and extensive thyroid tissue sections. Specifically, by using the IF technique, Fernandez-Santos et al. observed that approximately 76.09 ± 7.31% of thyrocytes in normal human thyroid tissue exhibited PCs, with an average ciliary length of 3.93 ± 0.90 μm (Fernández-Santos et al. 2019). These findings were consistent with those reported by Lee et al. (2019). Subsequently, the percentage of ciliated follicular cells was also investigated in relation to thyroid follicle size, as a potential indicator of their functional activity. However, this analysis revealed no statistically significant differences among small, medium, and large follicles (Fernández-Santos et al. 2019).

**Fig. 5:**
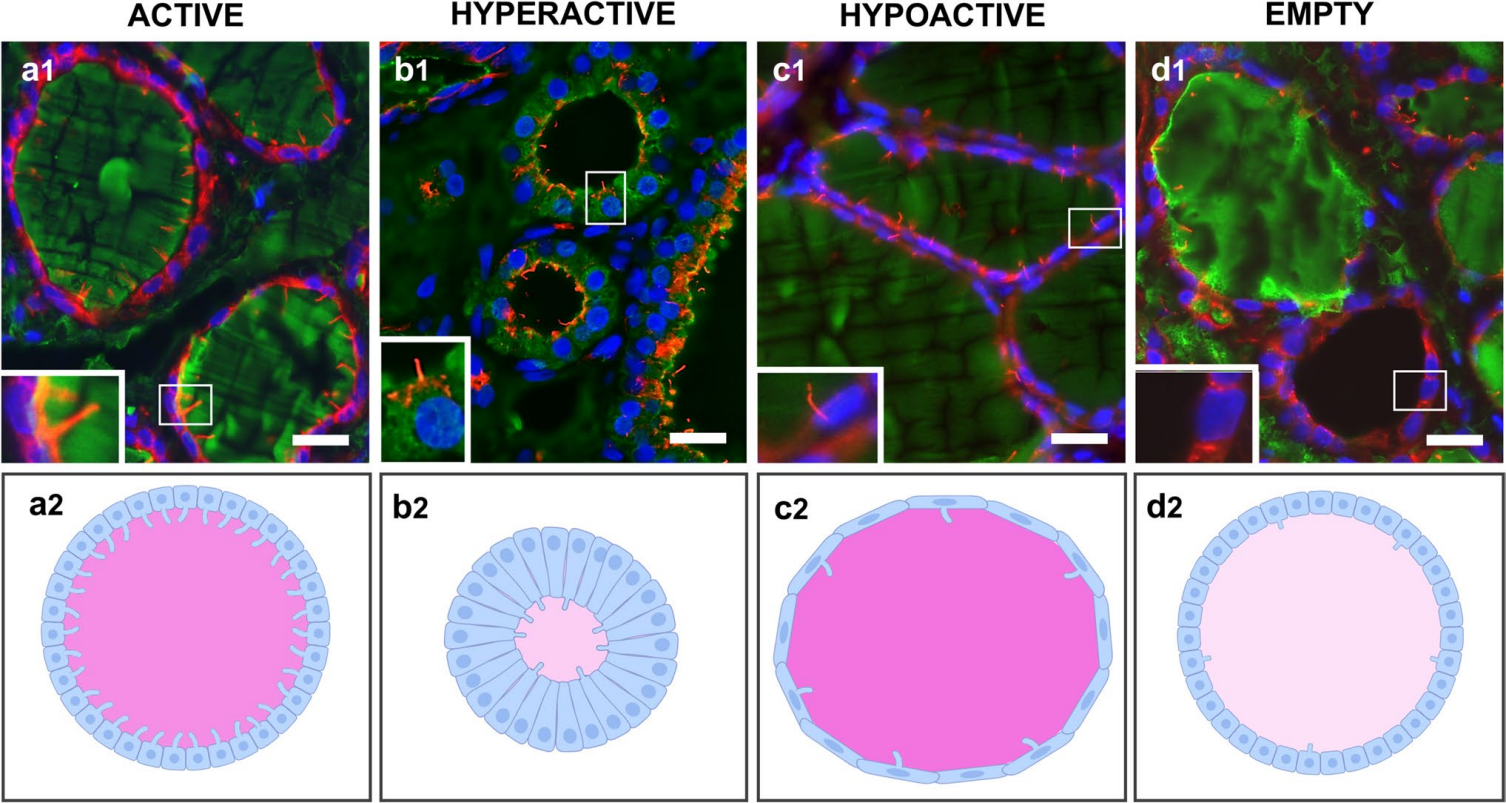
Identification of PCs by IF in different types of thyroid follicles (**a1**–**d1**) with their schematic representations below (**a2**–**d2**). Staining for Tg (green), PCs with acetylated α-tubulin (red), and nuclei with DAPI (blue): (**a1**) active follicles, (**b1**) hyperactive follicles, (**c1**) hypoactive follicles, and (**d1**) empty follicles. PC characteristics depend on the activity of thyroid follicles, being the most abundant and longest in active follicles. Scale bars 10 µm. Drawings made with Biorender

Therefore, a new approach was explored to investigate the potential relationship between PC and the TH biosynthetic process. Four types of thyroid follicles were characterized on the basis of their functional activity: active, hypoactive, hyperactive, and empty follicles (Pérez-Fernández et al. 2024). The results showed a significant variation in PC patterns among them, with active follicles exhibiting the highest percentage of ciliated thyrocytes, followed by hyperactive, hypoactive, and, notably less, empty follicles. Significant differences in PC lengths were also observed, being active follicles where the longest PCs were displayed, followed by hypoactive, hyperactive, and finally empty follicles. Hence, the characteristics of the PC were more conspicuous the more involved the follicles were in the TH biosynthetic process (Pérez-Fernández et al. 2024) (Fig. 5).

## Putative roles of the primary cilium in thyrocytes

### Primary-cilium-dependent signaling in thyrocytes

The PC has typically been associated with multiple intracellular signaling pathways, including sonic hedgehog (Shh), G protein-coupled receptor (GPCR), Wnt, class II/III receptor tyrosine kinases (RTKs; PDFGRα, insulin receptor, and EGFR), and TGF-β signaling (Anvarian et al. 2019), owing to the presence of receptors and effectors on the ciliary membrane, suggesting a role of PCs in recognizing extracellular ligands that modulate cellular function (Spasic and Jacobs 2017).

In the thyroid, PCs may be implicated in some of these pathways. Kim et al. associated Wnt signaling to thyroid-specific gene expression, since TSH-induced *Wnt-1* overexpression suppresses TPO transcription, allowing TSH to regulate cell growth and thyroid gene expression (Kim et al. 2007). Moreover, *WNT5A* and *TLR3* overexpression in papillary thyroid carcinoma highlights the relevance of this pathway for proper thyroid function (McCall et al. 2007).

The Shh pathway is aberrantly activated in thyroid cancer, suggesting PC involvement in thyroid tumorigenesis through this pathway (Xu et al. 2012). Furthermore, elevated RTK activity promotes RET/RAS/BRAF mutations, inducing noncanonical Shh activation, enabling PCs to integrate these pathways in the thyroid (Ma et al. 2021).

In addition, PC defects through TGF-β signaling can trigger epithelial–mesenchymal transition (EMT) and exacerbate autoimmune thyroid diseases, as demonstrated by Marazuela’s group when stimulating thyrocytes with TGF-β, which increased the expression of EMT markers and disrupted ciliogenesis (Sacristán-Gómez et al. 2023).

Through some of these pathways, the PC may function as a biosensor in the thyroid, potentially detecting the chemical composition of the colloid (Brix et al. 1996; Szumska et al. 2015; Qatato et al. 2018, 2021; Doğru et al. 2023) or sensing pressure changes resulting from lumen filling with Tg (Pérez-Fernández et al. 2024). Moreover, other potential roles have been proposed for the PC in this endocrine gland, including the relationship between ciliogenesis and autophagy (Lee et al. 2016b) or its involvement in the regulation of Tg endocytosis (Lee et al. 2021b), which may be mediated by classical signaling pathways.

### The primary cilium as a chemosensor in thyroid

 Trace amine associated receptor 1 (TAAR1), a GPCR involved in dopaminergic neurotransmission (Wolinsky et al. 2007; Revel et al. 2011, 2012; Harmeier et al. 2015) and a potential neuropsychiatric therapeutic target (Alvarsson et al. 2015; Grandy et al. 2016; Berry et al. 2017), has been investigated in the thyroid owing to the role of THs in brain development and neuropsychiatric diseases (Bauer et al. 2008). It has been detected in rodents and porcine thyrocytes (Brix et al. 1996; Szumska et al. 2015; Qatato et al. 2018), as well as in human KTC-1 (PTC) and Nthy-ori 3–1 (normal thyroid) cell lines (Qatato et al. 2021), suggesting a relevant role of this receptor in thyroid physiology. Specifically, it is concentrated in the apical membrane domain and the reticular vesicular system, and its localization in the PC of thyrocytes has been confirmed (Szumska et al. 2015; Qatato et al. 2018, 2021).

TAAR1 may participate in the detection of colloid chemical composition, as some thyronamines act as its ligands (Szumska et al. 2015). It also influences Tg proteolysis by cathepsins, as TAAR1 knockout (KO) reduces Tg degradation by altering protease activity. Furthermore, the activity of cathepsins may be associated with proper ciliogenesis in thyrocytes, as indicated by the colocalization of cathepsins L and B with TAAR1 in the PC of porcine thyrocytes. In fact, inhibiting cathepsins activity leads to TAAR1 delocalization in Follicular Rat Thyroid (FRT) cells, confining it to secretory pathway compartments, and almost complete loss of PCs (Qatato et al. 2018). A similar effect is observed in the Nthy-ori 3–1 cell line, where cathepsin inhibition reduces PC length (Qatato et al. 2021).

TAAR1 deficiency causes delocalization of the TSH receptor (TSH-R) from the basal membrane to intracellular vesicles, increasing serum TSH levels (Qatato et al. 2018) and reducing PC length, while raising cathepsin S expression. However, it remains unclear whether TSH affects the PC directly or through cathepsins (Doğru et al. 2023). Brix’s group showed that TAAR1 collaborates in maintaining the basolateral localization of TSH-R in vivo (Qatato et al. 2021), suggesting its role in the hypothalamus–pituitary–thyroid feedback loop detecting colloid chemical composition (Qatato et al. 2018).

### The primary cilium as a regulator of the endocytic pathway in thyrocytes

Tg endocytosis, a crucial step in TH biosynthesis, is mainly regulated by TSH, although other molecules are involved, among which is LRP2/megalin, a Tg receptor (Marinò et al. 2000b, 2000a, 2001; Marinò and McCluskey 2000). Lee’s group studied the PC’s role in Tg endocytosis using a mouse model of primary hypothyroidism induced by MMI. These mice showed irregular thyroid follicles with scant colloid, owing to the elevated rate of Tg endocytosis triggered by the overproduction of TSH in response to low serum TH levels. In addition, overexpression of genes related to endocytosis, including megalin, was observed on the apical surface of thyrocytes. MMI treatment also promotes ciliogenesis, linking it to Tg endocytosis, as confirmed by the colocalization of PCs with megalin (Lee et al. 2021b). In the nervous system, megalin mediates endocytosis and controls the Shh pathway (McCarthy et al. 2002; Ortega et al. 2012), which suggests that megalin mediates Tg endocytosis via Shh (Lee et al. 2021b).

### The primary cilium as a putative regulator of autophagy, homeostasis, and apoptosis of thyrocytes

The relationship between autophagy and the PC has been controversial, with some studies suggesting a positive regulation (Suzuki et al. 1999a, b, 2011), while others propose a negative regulation of primary ciliogenesis by autophagy (Pampliega et al. 2013). Lee et al. studied this interaction in Hürthle cells, characterized by mitochondrial defects, which showed enhanced autophagy and impaired PC formation owing to autophagic degradation of key ciliogenesis proteins. The conjunction of both characteristics is associated with poorer prognosis in thyroid tumors, as the absence of PCs is indicative of more dedifferentiated cells (Lee et al. 2016b). Collectively, these observations indicate an association between ciliogenesis and autophagy in thyrocytes, although the definitive proof of a causal relationship is still lacking.

In anaplastic thyroid carcinoma, an aggressive and dedifferentiated type of thyroid cancer, reduced PC frequency and length, as well as decreased expression of genes involved in ciliogenesis, have been observed. In this context, Lee’s group investigated the role of PCs in maintaining follicular architecture and homeostasis using a mutant mouse model with a loss-of-function (LOF) of IFT88. Loss of PCs in these mutants led to progressive thyroid follicle destruction and malignant transformation, emphasizing the crucial role of the PC in thyroid homeostasis and architecture (Lee et al. 2019).

Hürthle cell carcinoma, and other types of thyroid cancer, exhibit reduced primary ciliogenesis and mitochondrial abnormalities (Lee et al. 2016b), suggesting a potential link between PC alterations and the intrinsic apoptosis pathway. Lee et al. described in *Ift88* KO mice an enlarged and irregular appearance of thyroid follicles, with thyrocytes displaying apoptotic features, such as the downregulation of the anti-apoptotic protein *Bcl-2*. In thyroid carcinoma cell lines, knockdown of *KIF3A* or *IFT88*, along with PCs loss, increased apoptosis. Accordingly, PC dysfunction in thyroid tumor cells is a pro-apoptotic stimulus for the activation of intrinsic apoptosis (Lee et al. 2021a).

### The primary cilium as a mechanosensor in thyrocytes

The PC functions as a mechanosensor in cells exposed to mechanical forces, particularly in the kidney, via PC1/2, situated in the PC membrane of renal tubular cells (Spasic and Jacobs 2017). Other proteins involved in mechanotransduction, such as AC6 in cholangiocytes (Masyuk et al. 2006) and TRPV4 in osteocytes (Moore et al. 2018), have also been localized in the PC compartment.

In the thyroid gland, thyroid follicles exhibit variability in morphological parameters such as size, shape, follicular epithelial height, nuclear shape, and colloid density (Pérez-Fernández et al. 2024). This intrinsic heterogeneity of the thyroid gland suggests the need for self-regulation of each follicle. Specifically, Suzuki et al. proposed that Tg is the main regulator of thyroid-specific gene expression in each follicle, under the same general level of TSH, so that Tg acts as a negative feedback signal on the endocrine function and cellular growth of thyrocytes (Suzuki et al. 1999a, b, 2011).

**Fig. 6:**
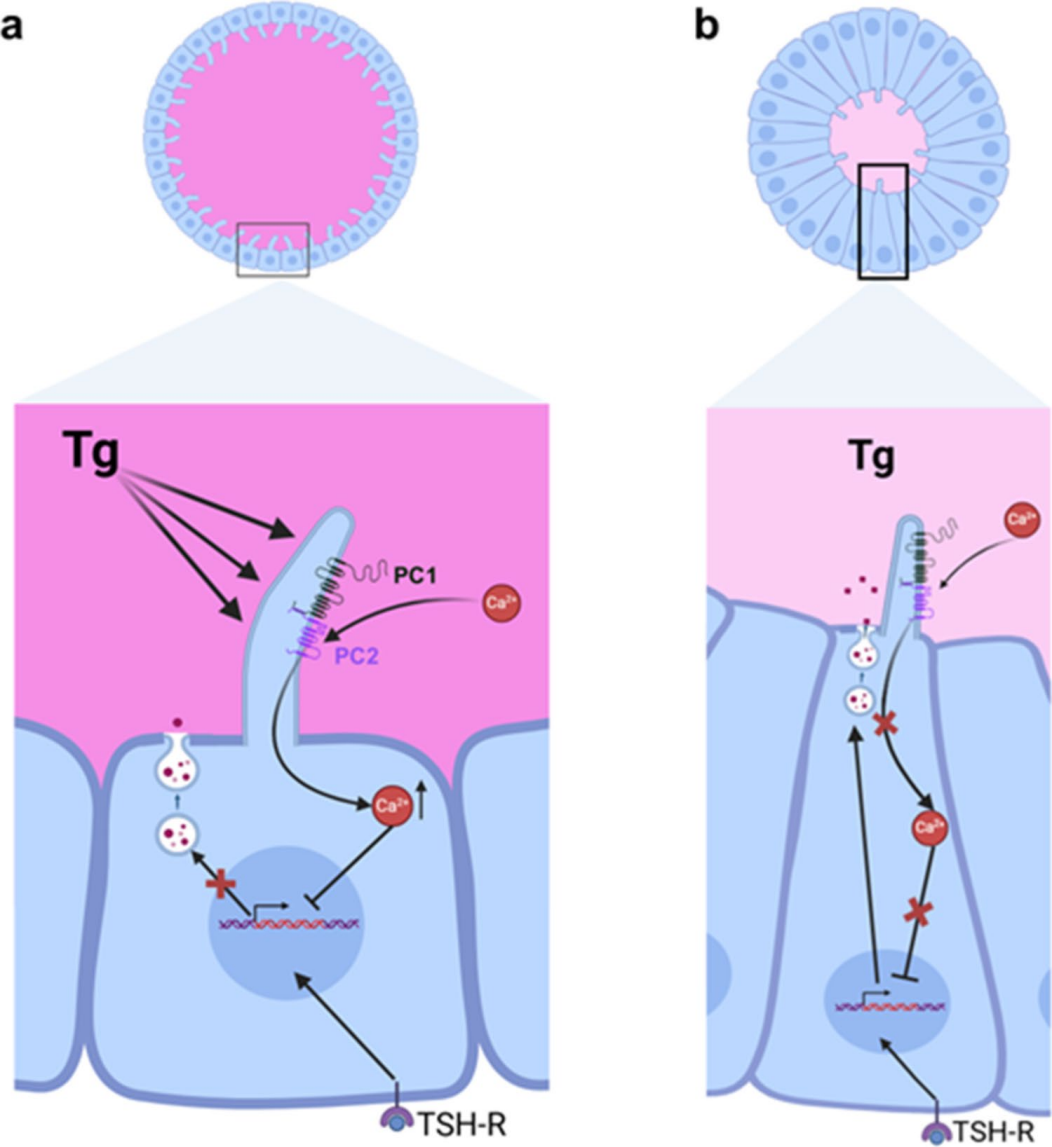
Schematic representation of changes in PC morphology in relation to thyroid follicle activity. In active follicles (**a**), the PC may mechanically sense the high colloid density, triggering the inhibition of thyroid-specific gene transcription and, ultimately, resulting in a decrease in Tg synthesis. Conversely, in hyperactive follicles (**b**), the lack of mechanical stimuli in the lumen promotes the reduction of the length of PCs, thus minimizing the negative feedback on Tg production and allowing full TSH stimulation of Tg biosynthesis. Made with Biorender

As mentioned above, several patterns of thyroid follicles were identified on the basis of their biosynthetic activity level, which is related to characteristics such as follicular epithelial height and colloid density (Pérez-Fernández et al. 2024). Variations in PC morphometry across the different types of follicles were observed, with active follicles showing more prominent PCs, reinforcing the connection between thyroid function and this organelle (Fig. 5) (Pérez-Fernández et al. 2024). With this evidence, it was suggested that the PC may act as a mechanosensor in thyroid follicles, detecting rises in lumen filling or colloid density and leading to the suppression of thyroid-specific gene expression. Conversely, in follicles with low Tg storage, the lack of mechanical stimuli minimizes the negative feedback on Tg synthesis, allowing full TSH stimulation of Tg biosynthesis (Pérez-Fernández et al. 2024). Thus, the role of the PC as a mechanosensor capable of detecting changes in Tg concentration may be the underlying mechanism driving the self-regulation of individual follicles mediated by the Tg stored in their own lumen and is in accordance with the Suzuki et al. proposal (Suzuki et al. 1999a, b, 2011). This process would provide a local, follicle-specific feedback system, allowing each follicle to autonomously adjust its activity in response to its intraluminal environment and thereby contributing to the characteristic heterogeneity observed in the thyroid gland (Fig. 6). Nevertheless, further functional studies are needed to validate the hypothesis of a mechanosensory role of PCs in the thyroid gland.

In summary, the PCs in the thyroid appear to be involved in various intracellular signaling processes and could be considered “cellular antennae” or biosensors for diverse stimuli, with their role in mechanotransduction being particularly promising.

## Primary cilia dysfunction in thyroid: does a specific thyrociliopathy exist?

As mentioned above, ADPKD is currently considered a systemic disease that, although primarily affecting the kidney, is accompanied by multiple extrarenal alterations. Among them, the most common are cysts in the liver, pancreas, spleen, and uterus (Yu et al. 2019). Typically, these complications begin to manifest in advanced stages of the disease, although their progression depends on individual factors (Higashihara et al. 2012).

However, chronic renal failure is considered a predisposing factor to alterations in the biosynthesis of THs, possibly owing to malnutrition, vitamin D deficiency, inflammation, or metabolic acidosis present in patients undergoing dialysis (Cuna et al. 2017). In these patients, the incidence of hypothyroidism accompanied by low serum levels of T_3_ increases compared with the general population, owing to the so-called Wolff–Chaikoff effect, in which the peripheral conversion of T_4_ to T_3_ is reduced by alterations in the renal excretion of iodine (Zalewska et al. 2022). It should be noted that functional alterations of the gland are accompanied by a higher incidence of benign and malignant thyroid nodules (Sanai et al. 2010).

Although the relationship between renal and thyroid dysfunction seems clear, several studies have attempted to assess whether ADPKD is associated with a higher incidence of nodules or cysts in the thyroid gland, as occurs in other organs. In this regard, conclusions are contradictory. While some studies have reported a significant incidence of polycystic thyroid or multiple nodules in patients with long-standing ADPKD (Alejmi and Sayer 2008; Pirson 2010), others have not found an increase in thyroid lesions in the early stages of the disease and, therefore, consider that the cause of these findings in chronic patients is due to the renal failure inherent to this pathology (Zalewska et al. 2022).

To the best of our knowledge, the investigation of ciliogenesis in thyroid tissues from patients with ADPKD remains unexplored. Similarly to what occurs in the kidney or liver, if alterations of thyroid ciliogenesis were confirmed in patients with ADPKD, the existence of a thyrociliopathy may be postulated. In this context, impaired PC sensory function would be postulated as the main triggering factor.

## Ciliogenesis in thyroid diseases

### Primary cilia in functional thyroid pathology

The most common cause of thyroid disorders worldwide is iodine deficiency, but even in relatively iodine-sufficient Western countries, according to the 2023 clinical practice guidelines of the European Thyroid Association (ETA), more than 60% of the adult population present one or more nodules in the thyroid gland (Durante et al. 2023).

In the last decade, the role played by the PC in thyroid disease has been explored in literature. Lee et al. reported no remarkable changes in either the frequency of ciliated cells or the cilia lengths in benign nodular hyperplasia (NH) when compared with those in normal thyroid glands. The authors cited follicular heterogeneity and structural variability of follicles, as a representative feature of this disease, which would account for the lack of differences observed in their study (Lee et al. 2016a). Our group studied the ciliogenesis process in thyroid glands with diffuse hyperplasia (DH) (from patients diagnosed with Grave’s disease) and NH (Fernández-Santos et al. 2019). Thyroid tissue from both DH and NH diseases exhibited changes in the frequency, distribution, and morphology of PCs, with lower ciliary frequencies and shorter axonemal lengths when compared with normal thyroid glands. Moreover, we observed a generally lower frequency of PCs in zones with altered follicles compared with normal appearing areas, suggesting that thyroid primary ciliogenesis is associated with functional pathology, but also and perhaps more importantly, with thyroid tissue heterogeneity.

Subsequently, we explored the ciliogenesis process in Pendred syndrome thyroid tissue (Vázquez-Román et al. 2022). Patients with Pendred syndrome exhibited a wide spectrum of thyroid pathological entities, ranging from NH and DH, multiple follicular adenomas to hyperplastic adenomatoid nodules. In accordance with our previous work, the frequency of ciliated cells and PC lengths varied among different areas in accordance with their follicular architecture. Thus, both parameters drastically decreased in highly cellular follicular nodules and follicular adenomas.

In summary, current evidence indicates that impaired ciliogenesis is associated with functional thyroid disorders. However, considering the intrinsic histological heterogeneity of the thyroid gland, alterations in ciliogenesis appear to be primarily linked to the histological features and activity of the individual thyroid AFUs, as defined by Colin et al. (Colin et al. 2013).

### Primary cilia in autoimmune thyroid disease

Autoimmune thyroid disorders (AITD), which include Hashimoto’s thyroiditis (HT) and Graves’ disease (GD), are the most common thyroid conditions in iodine-sufficient regions (Vanderpump 2011). They represent the most prevalent autoimmune diseases globally, affecting approximately 5% of the population (Weetman and DeGroot 2000; Mammen and Cappola 2021). Recent studies have explored the potential link between primary ciliogenesis and the pathogenesis of AITD (Lee et al. 2016b; Fernández-Santos et al. 2019; Martínez-Hernández et al. 2019; Sacristán-Gómez et al. 2023).

Lee et al. reported no differences in ciliogenesis between HT and normal thyroid tissues overall (Lee et al. 2016b). Furthermore, we found a general reduction in PCs frequency and length in thyroid tissues from patients with GD (Fernández-Santos et al. 2019); the findings corroborated by Martínez-Hernández et al. (2019). The latter also identified downregulation of several cilia-related genes (ENO4, INTU, KIF27, PACRG, and STK36) in both GD and HT. Contrary to previous reports, Martínez-Hernández et al. observed alterations in HT but not in NH, attributing these discrepancies to thyroid tissue heterogeneity. More recently, Sacristán-Gómez et al. (2023) demonstrated that TGF-β-treated thyrocytes exhibit increased mesenchymal marker expression and disrupted primary ciliogenesis, suggesting a potential role for EMT and PC dysfunction in AITD pathogenesis (Sacristán-Gómez et al. 2023).

### Primary cilia in thyroid neoplasms

Thyroid cancer is the most common endocrine malignancy, and its incidence and mortality have increased steadily over the last decades. Carcinomas derived from thyroid follicular cells can be divided into well-differentiated thyroid carcinomas (WDTC), with two main subtypes: papillary thyroid carcinomas (PTC) and follicular thyroid carcinomas (FTC); poorly differentiated thyroid carcinomas (PDTC); and anaplastic thyroid carcinomas (ATC). PTC is the most frequent form of WDTC, accounting for 80–85% of all thyroid carcinomas. FTC represents 10–15% of all thyroid carcinomas. Less common but highly aggressive forms are PDTC and ATC, with frequencies of 6% and 1–2%, respectively (Zaballos and Santisteban 2017). Most PTCs are associated with relatively good survival, even in the metastatic setting. However, there are some variants of PTCs with more aggressive behavior, such as the solid variant (Coca-Pelaz et al. 2020).

Oncogenic mutations coupled to specific intracellular downstream signaling pathways, mainly MAPK and PI3K pathways, lead to the development of different subtypes of thyroid cancer (Zaballos and Santisteban 2017). The PC, as potential mediator of these signaling pathways, is likely implicated in thyroid tumorigenesis. In fact, the first evidence of aberrant changes of PCs in human thyroid tumors was reported as early as 1987 by Nesland et al. Using SEM, they qualitatively described a gradual reduction in the number of PCs from normal thyroid tissue, through thyroid adenomas (follicular and Hürthle adenomas), and WDTC (FTC and PTC), ultimately becoming absent in ATC (Nesland et al. 1987).

Almost 30 years later, Lee et al. (2016b) examined the distribution of PCs in human thyroid cancer to address the involvement of abnormal ciliogenesis in thyroid tumorigenesis. Specifically, they described that the frequency of ciliated cells was comparable in different PTC variants, except for the oncocytic. By contrast, ciliogenesis was markedly decreased in Hürthle cell tumors and ATC, the more aggressive class of thyroid carcinoma (Lee et al. 2019).

In human follicular cell lines, Utrilla et al. found a lower frequency of PCs in neoplastic cells (FTC, 8505C) and, specifically, in ATC cells (Utrilla et al. 2015). These findings were corroborated by Lee’s group, whose study encompassed a more extensive panel of cell lines (Lee et al. 2016b, 2019). They reported that the frequency of PCs in PTC cells (TPC1/RET rearrangements and BCPAP/BRAF^V600E^) was not statistically significantly different from that in normal follicular cells (Nthy-ori 3–1), whereas the frequency of PCs in ATC cell lines (8505C, SW1736, and HTh7) was significantly lower than that in Nthy-ori 3–1 or PTC cell lines.

As commented previously, the same research group generated mice devoid of PCs by thyroid-cell-specific deletion of the gene encoding IFT88 (Lee et al. 2019). These mice showed structurally abnormal follicles and papillary-solid proliferative thyroid lesions, which eventually progressed to aggressive and dedifferentiated thyroid carcinomas. These findings indicate that loss-of-function of PCs contributes to malignant thyroid transformation (Lee et al. 2019).

More recently, Ma et al. described in BRAF^V600E^-positive human PTC samples a decrease in PCs that was associated with aggressive behaviors and with the number of PTC cells with the BRAF^V600E^ mutation. They also observed similar results in PTC cell lines and speculated that there was a negative correlation between the number of PCs and the number of PTC cells carrying the BRAF^V600E^ mutation (Ma et al. 2024).

Summarizing, the PC loss in thyroid cancer is more pronounced in carcinomas exhibiting the most aggressive behavior, particularly ATC, but also certain subtypes of PTC, such as the oncocytic variant and Hürthle cell carcinoma.

### Thyroid tumorigenesis, signaling pathways, and the primary cilium

As previously mentioned, several intracellular signaling pathways are involved in regulating thyrocyte activity and growth. Specifically, Wnt signaling has been shown to contribute to TSH’s ability to simultaneously increase cell growth and functional thyroid-specific gene expression in FRTL-5 cells (Kim et al. 2007). The Wnt/β-catenin pathway also plays a significant role in regulating the proliferation of primary human thyrocytes (Chen et al. 2010). In addition, alterations in this pathway, such as with Wnt5a, an effector of Wnt signaling, have been found to be overexpressed in thyroid cancer (McCall et al. 2007).

Similarly, Xu et al. (2012) have demonstrated that the Shh pathway is widely activated in thyroid neoplasms and may have potential as an early marker or a therapeutic target for thyroid cancer treatment. These authors showed that a Shh inhibitor primarily reduced the proliferation of WR082 and KAT-18 cells.

Another pathway commonly implicated in thyroid tumorigenesis is the MAPK and PI3K/AKT/mTOR pathway, whose overactivation has oncogenic characteristics (Zaballos and Santisteban 2017). Lin et al. (2017) tested a drug that inhibits the chaperone HSP90, which affects this pathway. Their in vivo experiments on ATC demonstrated that the drug promotes apoptosis and delays tumor growth. Similarly, Kim et al. (2020) used sodium selenite as an adjuvant to other MAPK pathway inhibitors, successfully reducing the proliferation of tumor follicular cell lines. Among the various signaling pathways mentioned above that operate in the thyroid, some require the presence of a PC for their activity. Specifically, Corbit et al. (2008) showed that the PC restricts the activity of the canonical Wnt pathway in various non-thyroid cells. Cell biological studies have also provided very strong evidence that mammalian Hh signaling depends on the PC (Huangfu and Anderson 2005). In contrast to the PC’s restrictive role on Wnt signaling, PC promotes Hh signaling, being present on Hh-responsive cells and act as organelles that are required for all activity of the mammalian Hh pathway (Huangfu and Anderson 2005). As both pathways are widely activated in thyroid neoplasms, it would be interesting to analyze the relationship between the expression of Wnt and Hh proteins with ciliogenesis in the most aggressive types of thyroid cancer.

### The primary cilium as a therapeutic target in thyroid cancer

Although certain tumors, such as medulloblastomas and basal cell carcinomas, require the presence of the PC for their development, most malignancies are characterized by a decrease or loss of PCs (Han et al. 2009; Hassounah et al. 2013; Gradilone et al. 2017). This includes cholangiocarcinoma, prostate cancer, ovarian cancer, renal cell carcinoma, pancreatic adenocarcinoma, melanoma, and, as this review now suggests, the most aggressive thyroid carcinomas. This collectively indicates that PC loss is a common occurrence in neoplastic transformation. It is plausible that the multisensory functions of the PC act as a brake on cell proliferation, migration, and invasion, and their loss during tumorigenesis gives an advantage to cancer cells to freely proliferate. Hence, restoration of the PC in cancer cells may represent a novel promising approach to attenuate tumor growth.

While the complete mechanisms underlying PC loss in diverse tumors have yet to be fully determined, Gradilone et al. provided a preliminary understanding through their analysis of ciliogenesis in human cholangiocarcinomas (Gradilone et al. 2013). They found that ciliary reduction in this neoplasia appeared to be linked to the overexpression of histone deacetylase 6 (HDAC6), an enzyme that deacetylates tubulin in the PC axoneme, thereby inducing their resorption (Gradilone et al. 2013). The same group reported a few years later a preclinical example of restoration of the PC as a possible therapeutic target in cholangiocarcinomas, by using HDAC inhibitors to reduce tumoral growth (Gradilone et al. 2017). The use of HDAC inhibitors has also been applied to treat different thyroid neoplastic cell lines, producing encouraging results in ATC cells (Spartalis et al. 2019). Nevertheless, their impact on primary ciliogenesis has not yet been reported.

As mentioned above, PCs have been implicated in several signaling pathways that are important for thyroid neoplastic progression, such as Hh, Wnt, and MAPK pathways, with the Hh signaling pathway being the best characterized. For example, cyclopamine (a Shh inhibitor) primarily reduced the proliferation of FTC and ATC neoplastic cell lines (Xu et al. 2012), although its effect on thyroid ciliogenesis remains unexamined.

In contrast to the effect on Hh signaling, PCs are normally associated with the suppression of the Wnt signaling pathway. Therefore, PC loss could play a role in the increased activation of Wnt signaling during thyroid tumorigenesis, acting as a tumor suppressor organelle, as it occurs in some prostate cancers (Peixoto et al. 2020; Hassounah et al. 2013). In thyroid carcinomas, a reduction in the proliferation of PTC cells was achieved using an inhibitor (phenylmethylthiazole) that reduced the overexpression of certain Wnt proteins; however, their effect on PCs was not studied (McCall et al. 2007).

Khan et al. (2016) screened a library of clinically evaluated compounds for their ability to restore PC expression in different non-thyroid neoplastic cell lines (pancreas, lung, kidney, and breast). They found several commonly used drugs (clofibrate, gefitinib, sirolimus, imexon, and dexamethasone) that restored ciliogenesis and attenuated cancer cell proliferation. Restoring PCs on thyroid cancer cells may be a potentially promising possible therapeutic strategy, as has been suggested by different authors (Ma et al. 2021; Tian et al. 2024).

## Conclusions and future directions

PCs are biosensors that perform diverse functions, depending on the cellular type and extracellular signals. In the human thyroid, PCs are located at the apical pole of the follicular epithelium, sensing the lumen’s environment. Given this strategic position, PCs are crucial for regulating TH biosynthesis and thyroid homeostasis, acting either as chemosensors, mechanosensors, or intervening in autophagy and endocytosis. Consequently, changes in thyroid function, whether physiological or pathological, are reflected in PC characteristics. Similarly, defects in primary ciliogenesis, or in the specific ciliary receptors involved in the regulation of TH biosynthesis, are expected to lead to pathological alterations of thyroid tissue, resulting in a possible specific ciliopathy or thyrociliopathy.

This review also highlights compelling evidence for the alteration and loss of the PC in thyroid cancer, particularly in more aggressive forms such as ATC. The interplay between the PC and crucial signaling pathways involved in thyroid tumorigenesis is emphasized. Considering the PC as a possible therapeutic target, investigations into drugs that restore ciliogenesis and attenuate cell proliferation warrant further promising research for their antineoplastic properties in thyroid cancer cells.

## Data Availability

No datasets were generated or analyzed during the current study.

## Abbreviations

AC6: Adenylate cyclase 6
ACIII: Adenylate cyclase III
ADPKD: Autosomal dominant polycystic kidney disease
AFU: Angiofollicular unit
AITD: Autoimmune thyroid disorders
ARL13B: ADP-ribosylation factor-like protein 13B
ATC: Anaplastic thyroid carcinomas
CFTR: Cystic fibrosis transmembrane conductance regulator
CSPP1: Centrosome and spindle pole associated protein 1
DH: Diffuse hyperplasia
DUOX 1/2: Dual oxidase 1/2
EMT: Epithelial–mesenchymal transition
FTC: Follicular thyroid carcinomas
GD: Graves’ disease
GPCR: G-protein coupled receptor
HDAC: Histone deacetylase
Hh: Hedgehog (pathway)
HT: Hashimoto’s thyroiditis
IF: Immunofluorescence
IFT: Intraflagellar transport
IHC: Immunohistochemistry
LRP2: Low density lipoprotein-related protein 2
MAPK: Mitogen-activated protein kinases
MMI: Methimazole
NH: Nodular hyperplasia
NIS: Na^+^/I^−^ symporter
PC: Primary cilium
PC1/2: Polycystin-1/2
PDTC: Poorly-differentiated thyroid carcinomas
PI3K: Phosphatidylinositol 3-kinase
PKD: Polycystic kidney disease
PTC: Papillary thyroid carcinoma
PTU: Propylthiouracil
RTK: Receptor tyrosine kinase
SEM: Scanning electron microscopy
Shh: Sonic hedgehog
SSTR3: Somatostatin receptor 3
TAAR1: Trace amine associated receptor 1
TEM: Transmission electron microscopy
Tg: Thyroglobulin
TGF-β: Transforming growth factor-β
TH: Thyroid hormone
TPO: Thyroperoxidase
TRPV4: Transient receptor potential cation channel, subfamily V, member 4
TSH: Thyroid stimulating hormone
WDTC: Well-differentiated thyroid carcinomas
